# Clinical strategies for acquired epidermal growth factor receptor tyrosine kinase inhibitor resistance in non-small-cell lung cancer patients

**DOI:** 10.18632/oncotarget.19925

**Published:** 2017-08-04

**Authors:** Lijun Dong, Dan Lei, Haijun Zhang

**Affiliations:** ^1^ Department of Oncology, Zhongda Hospital, Southeast University, Nanjing, China

**Keywords:** drug resistance, epidermal growth factor receptor, tyrosine kinase inhibitors, non-small cell lung cancer, chemotherapy

## Abstract

Epidermal growth factor receptor (*EGFR*) mutations (*EGFRm*^+^) occur in 10–35% of non-small-cell lung cancer (NSCLC) cases and confer sensitivity to EGFR tyrosine kinase inhibitors (TKIs). *EGFR* TKIs are standard treatments for NSCLC patients harboring *EGFR* exon 19 deletions or exon 21 L858R point mutations. Despite initial benefit, most patients develop drug resistance, posing a challenge to oncologists. The secondary T790M point mutation in *EGFR* exon 20 contributes to approximately 60% of resistance cases. Optimum strategies for overcoming acquired EGFR TKI resistance are not clearly defined, although current common practice is to switch to platinum-based chemotherapy following resistance onset. While the second-generation EGFR TKIs, including afatinib, dacomitinib, and neratinib, exhibit promising preclinical activity against T790M mutants, dose-limiting toxicities in patients have limited clinical success. However, third generation EGFR TKIs appear able to overcome this mutation. Other treatment options aimed at EGFR TKI resistance include use of an EGFR TKI beyond progression, and chemotherapy plus an EGFR TKI. This review focuses on improved anticancer agents and therapy options for NSCLC patients with acquired EGFR TKI resistance.

## INTRODUCTION

Lung cancer is the leading cause of cancer-related mortality worldwide, and non-small-cell lung cancer (NSCLC) accounts for 80–85% of all lung cancer cases [1]. Epidermal growth factor receptor (*EGFR*) mutations (*EGFR*m^+^), such as exon 19 deletions and exon 21 L858R point mutations, occur in 10–35% of NSCLCs and confer sensitivity to EGFR tyrosine kinase inhibitors (TKIs) [2, 3]. *EGFR*m^+^ NSCLC patients benefit from treatment with first-generation EGFR TKIs (reversible, not mutant selective), such as erlotinib and gefitinib, which are the standard first-line therapy for these patients. However, most patients develop drug resistance within approximately one year of treatment due to various mechanisms [4, 5]. The secondary T790M point mutation in exon 20 contributes to approximately 60% of resistance cases. Additionally, about 30% of *EGFR*m^+^ patients show primary resistance to EGFR TKIs, and the factors involved in de novo resistance remain unidentified. Although the second-generation EGFR TKIs (irreversible, potent, not mutant selective), including afatinib, dacomitinib, and neratinib, exhibited promising preclinical activity against T790M mutants, dose-limiting toxicities in patients inhibited clinical success. Therefore, optimum treatments after disease progression resulting from acquired EGFR TKI resistance are not clearly defined. Switching EGFR TKI resistant patient treatment strategies to platinum-based chemotherapy is common practice. Fortunately, third-generation EGFR TKIs (mutant selective), such as osimertinib, rociletinib, and olmutinib, can overcome resistance resulting from T790M mutation [6–8]. For example, in 2015, osimertinib was approved for T790M-positive patients who had progressed on prior systemic therapy. Other treatment options aimed at EGFR TKI resistance include use of an EGFR TKI beyond progression, and chemotherapy plus an EGFR TKI. Since drug resistance to EGFR TKIs is a major clinical obstacle, and optimum treatment strategies are still elusive, this review focuses on anticancer agents and treatment options aimed at EGFR TKI resistant cases to better guide oncologists making clinical decisions.

## MECHANISMS OF ACQUIRED RESISTANCE TO EGFR TKIS

Acquired resistance to EGFR TKIs in NSCLC patients is defined as disease progression after a period of clinical benefit, and is categorized into three clinical groups: central nervous system sanctuary progressive disease (PD); oligo-PD; and systemic PD [9, 10]. The mechanisms involved can include secondary mutations in *EGFR*, bypass or alternative pathway activation, and histological and phenotypic transformations (Table 1). Several acquired-resistance mechanisms and candidates could also act as both rational targets and biomarkers. The best studied of these is the T790M mutation in exon 20. Additionally, *MET* mutations and amplifications, and amplifications of the MET ligand are also potential predictive biomarkers for NSCLC patient treatment outcomes, and insulin-like growth factor-1 receptor (IGF-1R) is a biomarker for TKI resistance [11, 12].

**Table 1:** Main mechanisms involved in acquired resistance to EGFR TKIs

Mechanism	Molecular alteration
**Secondary mutations in *EGFR***	T790M
	A761T
	T854A
	L747S
**Bypass or alternative pathway activation**	*MET* amplification
	*PIK3CA* mutation
	*HER2* amplification
	*BRAF* mutation
	*AXL* activation
**Histological and phenotypic transformation**	NSCLC to SCLC
	epithelial to mesenchymal

### Secondary mutations in *EGFR*

Secondary EGFR mutation is the most frequent mechanism of acquired resistance to EGFR TKIs. The secondary point mutation, T790M in exon 20, contributes to approximately 60% of drug resistance cases. [4, 5]. With the steric hindrance of the bulky methionine sidechain, T790M restores the kinase's adenosine triphosphate (ATP) affinity back to wild type levels, eventually re-establishing ATP as the favored substrate rather than the EGFR TKI [13]. Other rare EGFR point mutations resulting in resistance include, A761T, T854A, and L747S [14–16].

### Bypass or alternative pathway activation

MET signaling activates various pathways, such as PI3K/AKT, RAS-RAF-ERK1/2, and STAT3, promoting angiogenesis and tumor cell growth, survival, migration, invasion, and metastasis [11]. *MET* amplification, identified in 5–10% of resistant tumors, is the main mechanism by which EGFR inhibition is bypassed, allowing for drug resistance via ERBB3-mediated activation of PI3K/AKT downstream signaling [17]. Other bypass mechanisms include, *PIK3CA* mutation, *HER2* amplification, *BRAF* (V600E, G469A) mutation, and *AXL* activation [18–20].

### Histological and phenotypic transformation

After an initial response to EGFR TKIs, *EGFR*m^+^ NSCLC can transform to small cell lung cancer (SCLC) [18]. Epithelial to mesenchymal transition (EMT), which renders cells more migratory and invasive, is also observed in EGFR TKI-resistant tumor specimens [21]. EMT may be induced and controlled by multiple pathways, including Wnt, NFκB, and TGF-β signaling, as well as growth factors, such as FGF and EGF. Jakobsen, *et al*. found that erlotinib increased IGF1R activation, restoring PI3K/AKT and MAPK signaling, and triggering IGF1R-dependent EMT [22]. Alternatively, cancer cells treated with erlotinib may benefit from TGF-β signaling, which initiates EMT by inhibiting the miR-200 family, upregulating mesenchymal transcription factors and thus the EGFR repressor, MIG6, and restoring PI3K/AKT signaling. EMT is likely important in the development of NSCLC acquired EGFR TKI resistance, and influences TKI treatment response. EMT status may therefore be useful as a prognostic indicator and in treatment strategy decision-making in NSCLC.

## CLINICAL STRATEGIES FOLLOWING EGFR TKI ACQUIRED RESISTANCE IN NSCLC

**Figure 1:**
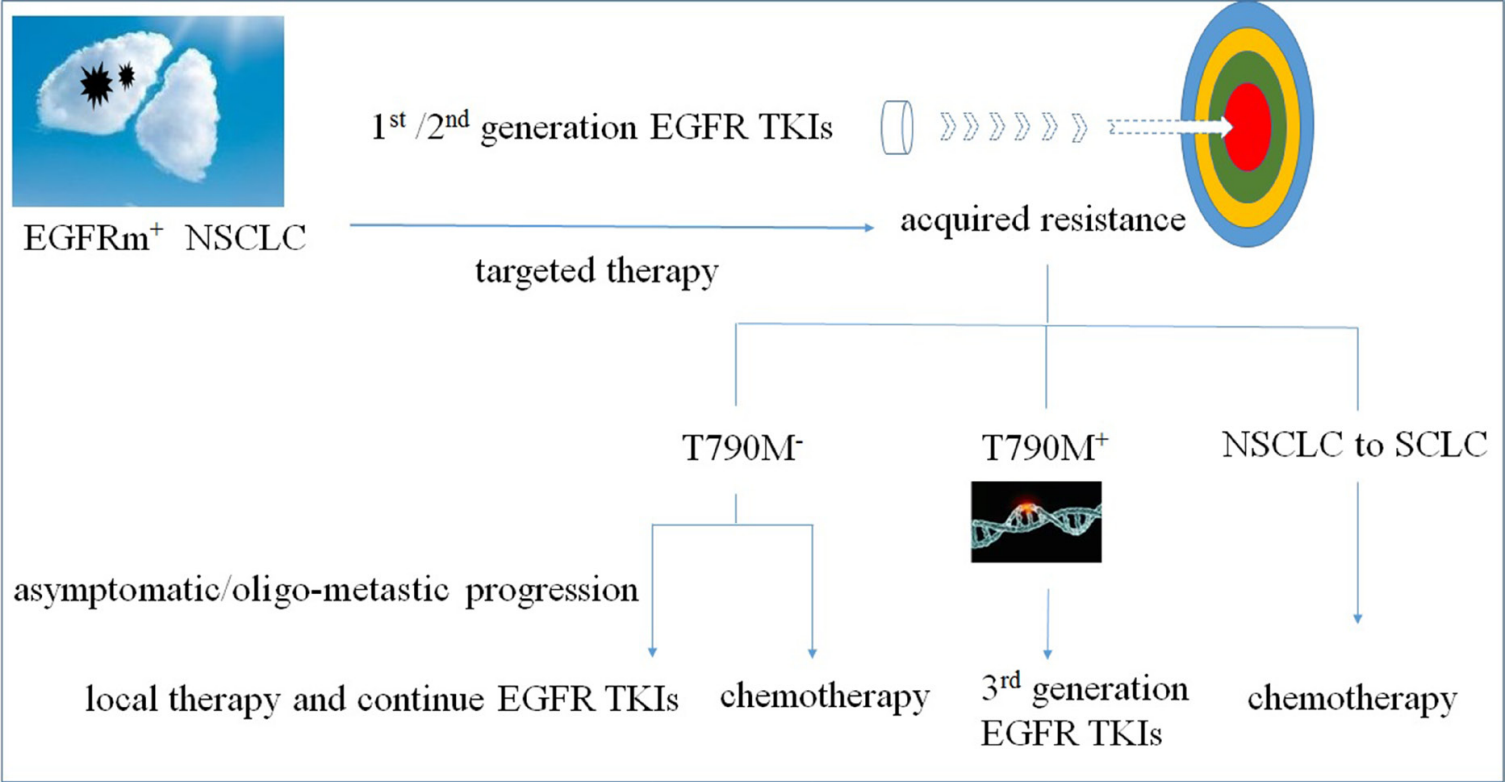
Treatment options for NSCLC patients with EGFR TKI acquired resistance

There are currently no standardized clinical strategies for NSCLC patients with EGFR TKI acquired resistance. Treatment options usually include chemotherapy, third generation EGFR TKIs, EGFR TKI treatment beyond progression, and chemotherapy plus an EGFR TKI (Figure 1).

### Chemotherapy

When first-line EGFR TKI acquired resistance causes clinical progression, NSCLC patients are commonly switched onto platinum-based chemotherapeutics, which often provide palliative benefit. Second-line platinum-based combination therapies led to better overall survival (OS) than single agents or erlotinib, following the failure of first-line gefitinib treatment [23]. Second-line gemcitabine/platinum therapy combination treatment resulted in better OS in *EGFR*m^+^ patients (*p* = 0.035), but not in those with wild-type *EGFR* (*p* = 0.785) [23]. Additionally, chemotherapy can allow for simultaneous re-challenge with a TKI; previously arrested TKI-sensitive cells may repopulate more quickly than TKI-resistant cells, and may therefore be efficiently inhibited using TKIs [24].

### Third-generation EGFR TKIs

**Figure 2:**
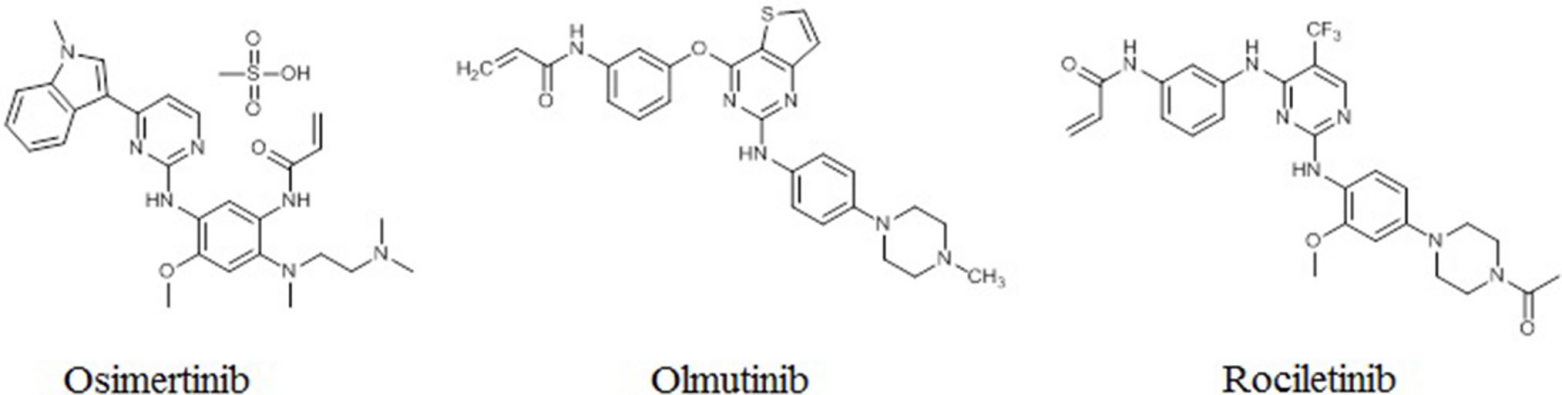
Structures of third-generation EGFR TKIs

*EGFR*m^+^ NSCLC patients can benefit from treatment with first-generation EGFR TKIs, including erlotinib and gefitinib [25, 26]. However, most patients develop drug resistance, driven in about 60% of cases by the T790M mutation [27, 28]. Second-generation EGFR TKIs cannot overcome T790M-mediated drug resistance due to nonselective inhibition of wild-type EGFR [6, 29, 30]. More recently, third-generation EGFR TKIs, including osimertinib, rociletinib, and olmutinib, have shown efficacy against the T790M mutation while sparing wild-type EGFR *in vitro* (Figure 2) [31, 32]. The third-generation EGFR TKIs represent a promising approach to overcoming T790M-mediated resistance to first- and second-generation EGFR TKIs in NCSLC patients.

### Osimertinib

Osimertinib (AZD9291; AstraZeneca) exhibits 200-fold greater selectivity for the T790M/L858R EGFR than wild-type EGFR [33]. It has been hailed as a “breakthrough” compound with perfect objective response rate (ORR) in T790M-positive NSCLC patients who had progressed on first-generation EGFR TKIs [34]. Preclinical studies confirmed osimertinib antitumor activity and reduced activity against wild-type EGFR in cell lines and tumor xenograft and transgenic mouse models harboring *EGFR*m^+^ and *EGFR* T790M [35]. The osimertinib ORR in the phase I clinical trial was 51%. In patients with confirmed *EGFR* T790M (*n* = 127), osimertinib had an ORR of 61% and median progression-free survival (mPFS) of 9.6 months, while T790M-negative patients (*n* = 61) had an ORR of 21% and mPFS of 2.8 months. [36] A pooled analysis of two phase II studies found that osimertinib ORR was 66% in T790M patients. Median duration of remission (DOR) was 12.5 months, and mPFS was 11.0 months. At 12 months, 47.5% of patients were progression-free [37]. Osimertinib was approved in the US on November 13, 2015 for use in patients with metastatic EGFR T790M-positive NSCLC who have progressed with EGFR TKI therapy.

### Rociletinib

Rociletinib (CO-1686) is another small-molecule, irreversibly binding, mutant selective TKI that targets commonly mutated forms of EGFR while sparing the wild-type protein [38]. In a phase I/II study of rociletinib in *EGFR*m^+^ NSCLC patients who had progressed during EGFR TKI therapy, ORR and mPFS were 59% and 13.1 months, respectively, in 46 T790M-positive patients, and 29% and 5.6 months, respectively, in 17 T790M-negative patients. Unlike for earlier generation EGFR TKIs, treatment side effects, including paronychia, rash, stomatitis, and diarrhea, were uncommon in rociletinib-treated patients. One patient experienced a grade 1 rash, and grade 1–2 diarrhea was observed in only 20% of patients [39].

### Olmutinib

Like osimertinib and rociletinib, olmutinib (HM61713) is a third-generation EGFR TKI that selectively targets mutant EGFR. In a phase I/II study, olmutinib ORR was 58.8% and the disease control rate was 97.1% in 34 T790M-positive NSCLC patients [40].

### Chemotherapy plus EGFR TKI

Patients with acquired resistance to EGFR TKIs may benefit from continued EGFR TKI therapy in combination with platinum-based chemotherapy. In a randomized, phase III, multicenter study, *EGFR*m^+^ patients with acquired resistance to gefitinib were randomly assigned (1:1) to either continued gefitinib treatment combined with pemetrexed/cisplatin chemotherapy or placebo plus pemetrexed/cisplatin chemotherapy. Ninety-eight (74%) patients progressed in the gefitinib plus chemotherapy group, and 107 (81%) progressed in the placebo plus chemotherapy group (hazard ratio = 0.86, 95% CI = 0.65–1.13; *p* = 0.27). mPFS was 5.4 months in both treatment groups (95% CI = 4.5–5.7 with gefitinib and 4.6–5.5 with placebo). The results demonstrated that continuing gefitinib with chemotherapy after disease progression with gefitinib did not prolong patient PFS [41]. Additionally, continued gefitinib treatment with chemotherapy may be detrimental to patient OS, although these findings require further study [41].

### Combined EGFR signaling pathway blockade

Targeting several levels of EGFR signaling is another strategy employed against acquired resistance to EGFR TKIs. Afatinib combined with the monoclonal anti-EGFR antibody, cetuximabhas, demonstrated robust clinical activity and a reasonable safety profile in patients with acquired gefitinib or erlotinib resistance, both with and without T790M mutations [42]. As previously discussed, bypass or alternative pathway activation can contribute to acquired resistance to EGFR TKIs. Thus, treatment strategies that both maintain inhibition of EGFR signaling and inhibit bypass signaling may be efficacious against resistance. EGFR TKIs combined with the aPI3K inhibitor, buparlisib (BKM120), or MET inhibitors, such as cabozantinib, tivantinib, or INC280, are presently under investigation for use in resistant NSCLC cases [42, 43].

### TKIs beyond progression

Discontinuing TKI treatment may lead to a disease flare [45]. In cases of isolated progression, such as in the central nervous system (CNS), local therapy plus targeted therapy may be efficacious in some patients [46, 47]. In patients with CNS metastasis, controlling brain lesions with radiotherapy is important for maintaining or improving quality of life and for prolonging survival [48]. The phase II, single-arm, open-label ASPIRATION (Asian Pacific trial of Tarceva as first-line in EGFR mutation) study investigated post-progression continued erlotinib therapy in NSCLC patients with activating *EGFR* mutations [49]. In this study, patients received erlotinib until progression, after which erlotinib was continued if deemed appropriate. Patients who received post-progression erlotinib therapy had longer PFS, longer time from best overall response to progression, fewer new lung lesions, and better performance status at progression. The ASPIRATION trial showed that in clinically asymptomatic patients, maintaining EGFR TKI therapy as long as possible is a viable treatment option.

To effectively provide the best treatment options for each patient, the mechanisms underlying resistance onset must be better understood. Analyses of tumor samples from NSCLC patients with acquired EGFR TKI resistance could reveal molecular mechanisms of resistance. Thus, performing a biopsy at the time of acquired resistance may inform treatment decisions [50, 51]. Because procedures used to obtain tumor tissues are often invasive, analysis of circulating plasma cell-free DNA (cfDNA), which is released from tumors or circulating tumor cells and can be collected noninvasively, could provide tumor cell genetic information in many cases [52, 53].

### Immunotherapy

Interest in immunotherapies for NSCLC treatment continues to grow. Preclinical evidence suggests that programmed death-ligand 1 (PD-L1) expression in *EGFR*m^+^ NSCLC may be constitutively driven by EGFR signaling. Preclinical *EGFR*m^+^ lung cancer models treated with PD-L1 antibodies exhibited reduced tumor growth and increased survival [54]. However, clinical data suggest that anti-PD1 immunotherapy may not be effective in *EGFR*m^+^ NSCLC.

## CONCLUSIONS

Optimum treatment strategies for NSCLC patients with acquired resistance to EGFR TKIs are currently unclear. This review summarized anticancer agents and clinical strategies employed following EGFR TKI acquired resistance onset in NSCLC patients, emphasizing therapeutic advances with improved outcomes. Current common practice is to switch to platinum-based chemotherapy following resistance onset. However, third-generation EGFR TKIs may overcome T790M-mediated resistance to first- and second-generation EGFR TKIs in NCSLC patients. In asymptomatic/oligo-metastic progression cases, localized therapies followed by continuation of EGFR TKI treatment may be a viable strategy.
